# Gerontology in Public Health: A Scoping Review of Current Perspectives and Interventions

**DOI:** 10.7759/cureus.65896

**Published:** 2024-07-31

**Authors:** Nor Faiza Mohd. Tohit, Mainul Haque

**Affiliations:** 1 Department of Community Health, Universiti Pertahanan Nasional Malaysia (National University of Defence Malaysia), Kuala Lumpur, MYS; 2 Department of Research, Karnavati Scientific Research Center (KSRC) School of Dentistry, Karnavati University, Gandhinagar, IND; 3 Department of Pharmacology and Therapeutics, Universiti Pertahanan Nasional Malaysia (National University of Defence Malaysia), Kuala Lumpur, MYS

**Keywords:** technology in health, mental health, caregiver support, preventive health, social determinants, health promotion, chronic disease, aging population, public health, gerontology

## Abstract

The intersection of gerontology and public health is increasingly vital due to the global aging population and its implications for health systems. This scoping review aims to map existing literature on gerontology within public health, identify current perspectives, and evaluate interventions tailored to the needs of older adults. A systematic search was performed using predefined keywords across multiple databases, including PubMed, Google Scholar, Scopus, and Web of Science. The review included 42 studies that employed various designs, all focusing on public health interventions targeting the aging population.

Key findings indicate a pressing need to integrate gerontological principles into public health practice, recognizing the heterogeneous nature of older adults and the significance of social determinants of health. Interventions ranged from preventive health measures and chronic disease management programs to health promotion activities and caregiver support, including the application of technology to improve health outcomes. However, there was a notable lack of research on diverse populations and mental health interventions. The review also uncovered critical gaps in the literature, such as economic barriers to care access and the necessity for comprehensive policies addressing the aging population's diverse needs.

In conclusion, this review emphasizes the importance of a multidimensional approach to effectively addressing older adults' health needs. While several effective interventions exist, there is an urgent need to tackle identified gaps, particularly concerning diverse populations and mental health, to enhance overall health strategies for the aging demographic.

## Introduction and background

The convergence of gerontology and public health is an increasingly crucial study area, especially in light of the growing aging population worldwide. According to the World Health Organization, the number of people aged 60 years and older will double from 900 million in 2015 to two billion by 2050 [1]. This unprecedented demographic shift poses significant global challenges and opportunities for public health systems, demanding a coordinated and comprehensive response. Gerontology, the study of the aging process and the issues that affect older adults examines various aspects of aging, including biological, psychological, and social dimensions [2,3]. In contrast, public health focuses on protecting and improving the health of communities through education, policymaking, and research for disease and injury prevention [4]. Integrating gerontological perspectives into public health frameworks is essential for developing effective policies and interventions that address the complex needs of older adults [5].

Historically, public health efforts have primarily targeted infectious disease control, maternal and child health, and chronic disease prevention and management. However, the increasing proportion of older adults necessitates a shift in focus towards geriatric public health [6]. This includes promoting healthy aging, preventing disease, managing chronic conditions, and enhancing the quality of life for older adults. Beard and colleagues (2015) assert that a comprehensive approach to healthy aging is needed, informed by a solid understanding of gerontology [7]. Aging is accompanied by various challenges, such as a heightened risk of chronic diseases, diminished functional capacities, and increased susceptibility to mental health issues, including depression and cognitive decline [8-10]. Furthermore, social determinants of health, such as economic status, housing conditions, and social isolation, significantly influence health outcomes in older populations [9,11,12]. These determinants necessitate a multifaceted approach to public health strategies, addressing medical and social needs.

In recent years, public health interventions have increasingly focused on the aging population's needs. These interventions include preventive health measures, chronic disease management programs, health promotion activities, and supportive services for caregivers. For instance, fall prevention programs have shown effectiveness in reducing the incidence of falls among older adults, enhancing their quality of life, and reducing healthcare costs [13]. Similarly, health promotion initiatives aimed at encouraging physical activity, healthy eating, and social engagement have been beneficial in fostering healthy aging [14,15]. Despite these advancements, significant gaps remain in understanding and implementing gerontological principles within public health practice. Research often lacks a comprehensive approach, failing to account for the diversity within the aging population. There is a dearth of studies focusing on minority groups, economically disadvantaged older adults, and other marginalized populations.

Additionally, mental health interventions targeting older adults are comparatively understudied, and there is a need for more robust evidence to inform effective policy and practice. For example, while cognitive-behavioral therapy (CBT) is widely used for younger populations, studies examining its effectiveness for older adults are limited. Research demonstrates that older adults often present with different symptoms of depression, such as increased irritability or cognitive decline, yet interventions frequently do not account for these unique factors [5,16,17].

Furthermore, integrating technology in health interventions for older adults presents opportunities and challenges. Telehealth services can improve access to mental health care, allowing older adults to receive therapy from home, especially those with mobility issues. However, the digital divide poses a significant barrier; many older adults may lack the necessary technological skills or access. For instance, a study found that only 42% of adults aged 65 and older use smartphones, limiting their ability to engage with telehealth platforms [18,19]. Addressing this digital divide through community-based training programs is crucial for ensuring equitable access to health interventions, allowing older adults to benefit fully from technological advancements in healthcare.

**Figure 1:**
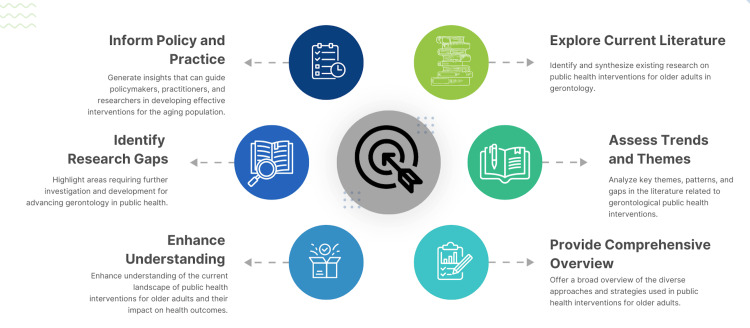
The objectives of the study. Image Credit: Nor Faiza Mohd. Tohit

This scoping review aims to map the existing literature on gerontology in public health, identify current perspectives, and evaluate the interventions implemented to address the needs of older adults. By synthesizing these findings, the review seeks to highlight both the progress made and the gaps that persist, thereby informing future research, policy development, and public health practice. This comprehensive understanding is imperative for creating compelling, inclusive, and sustainable health strategies that address the complex and diverse needs of the aging population (Figure 1). Several studies and data are needed to fill the gaps identified in the scoping review of gerontology in public health.

First, longitudinal studies that track the health outcomes of older adults over time would provide insight into the effectiveness of various interventions and the long-term impacts of public health strategies. These studies could assess how different factors, such as socioeconomic status or access to healthcare services, influence health outcomes in older populations. Second, qualitative research is essential to understanding older adults' lived experiences and perceptions regarding current public health interventions. Focus groups or interviews can reveal barriers to accessing services and highlight this demographic's specific needs and preferences, ensuring that future interventions are more tailored and effective. Third, randomized controlled trials (RCTs) evaluating specific interventions aimed at older adults, such as community-based health programs or technology-assisted health services, are crucial. These studies would provide robust evidence of what works, allowing for data-driven policy development and resource allocation. Lastly, data disaggregation by age, gender, ethnicity, and socioeconomic status is necessary to identify disparities within the aging population and ensure that public health strategies are inclusive and equitable. These research approaches will collectively enhance the understanding of older adults' needs, informing the development of effective, sustainable health strategies.

## Review

Methods and materials

This scoping review was conducted using the framework proposed by Arksey and O'Malley (2005), which involves identifying the research question, relevant studies, study selection, charting the data, and collating, summarizing, and reporting the results [20]. The study selection, extraction, and analysis processes concentrated on intervention types, populations studied, and critical outcomes guided by the Arksey and O'Malley framework [20]. Additionally, Arksey and O’Malley [20] divided it into six substructures to facilitate future authors in preparing the scoping review process. Those included: a) point out the study query, b) ascertain pertinent scientific paper, c) hand-picked studies, d) organize the necessary information, e) synopsize, combine, and comment on the results and f) comprise professional discussion. This framework was chosen for its systematic yet flexible approach, allowing for a broad exploration of the literature. The primary research questions were, e.g., to discuss the current perspectives on gerontology within the field of public health, to describe the interventions being implemented to address the health needs of older adults, and to address the gaps and challenges identified in integrating gerontology and public health.

A comprehensive literature search was conducted across multiple electronic databases, including PubMed, Google Scholar, Scopus, and Web of Science. The search strategy incorporated a combination of keywords and Medical Subject Headings (MeSH) terms related to gerontology, public health, aging population, and interventions. The search terms included "gerontology," AND "public health," AND "aging population," AND "disease prevention," AND "health promotion," AND "chronic illness," AND "social determinants," AND "elder care," AND "health outcomes," AND "preventive measures."

The Inclusion and Exclusion Criteria

The inclusion criteria were published in peer-reviewed journals, focused on populations aged 60 years and older, addressed public health perspectives or interventions related to gerontology, were written in English, and were published between 2000 and 2023 to ensure the relevance and currency of the findings.

The exclusion criteria were focused solely on clinical or medical treatments without a public health perspective, addressed populations younger than 60 years of age, not available in English, and studies focused on specific diseases or conditions not generalizable to broader public health or gerontological contexts. Review articles, editorials, commentaries, or opinion pieces without original research data were also excluded. 

Study Selection Process

The study selection involved multiple stages. First, the titles and abstracts of all identified articles were screened for relevance based on the inclusion and exclusion criteria. Two independent reviewers carried out this initial screening, ensuring objectivity and consistency. Studies deemed potentially relevant were then retrieved in full text for further assessment. Reviewer discrepancies were resolved through discussion and consensus or by consulting a third reviewer. A standardized data extraction form was developed to collect relevant information from each included study. The excluded data included a) Study characteristics (e.g., author, year, country, study design); b)Population characteristics (e.g., age, gender, socioeconomic status); c) Public health perspectives (e.g., attitudes, beliefs, policy implications); d)Interventions (e.g., type, duration, outcomes); and e)Key findings and recommendations.

The data were charted into a narrative summary and tabulated to facilitate a clear and comprehensive synthesis of the findings. The collated data were summarized thematically to identify key insights and trends in the literature. The analysis aimed to highlight the current perspectives on gerontology in public health, examine the effectiveness and scope of interventions, and identify gaps and areas for future research. Descriptive statistics were used where appropriate to provide an overview of study characteristics. Biases in scoping reviews can emerge at various stages, from study selection to data extraction and interpretation (Table 1). All the necessary strategies were carried out to overcome the possible biases by following Arksey and O’Malley [20] guidelines. 

**Table 1:** Type of biases and the strategies taken to overcome them.

Type of Bias	Strategy to Overcome
Selection Bias This can occur if studies are selectively included based on the reviewers' specific criteria or biases.	Implementing explicit inclusion and exclusion criteria is essential. Two independent reviewers conducted the initial screening and full-text assessment to minimize subjective judgment in study selection. Any discrepancies were resolved through consensus or consulting a third reviewer to ensure a balanced selection.
Publication Bias: Studies with positive results are more likely to be published, so there is a risk of overrepresenting them.	The search included multiple databases to capture a comprehensive range of studies, including those with negative or inconclusive results.
Reporting Bias: This refers to a skewed representation of results depending on how studies report their findings.	Ensuring a systematic approach to data extraction, where specific data points are consistently recorded, helps mitigate this bias. The use of standardized data extraction forms ensures consistency.
Language Bias: This occurs when only studies published in a particular language are included, potentially missing relevant studies in other languages.	Although this review included only English-language studies (a limitation acknowledged in the review), the search was exhaustive across several databases to capture a wide range of pertinent studies.
Reviewer Bias: The personal beliefs and experiences of the reviewers can influence the review process.	The reviewers who have diverse backgrounds and expertise can help mitigate this bias. Regular meetings and discussions were held to ensure an unbiased interpretation of the data.

**Figure 2:**
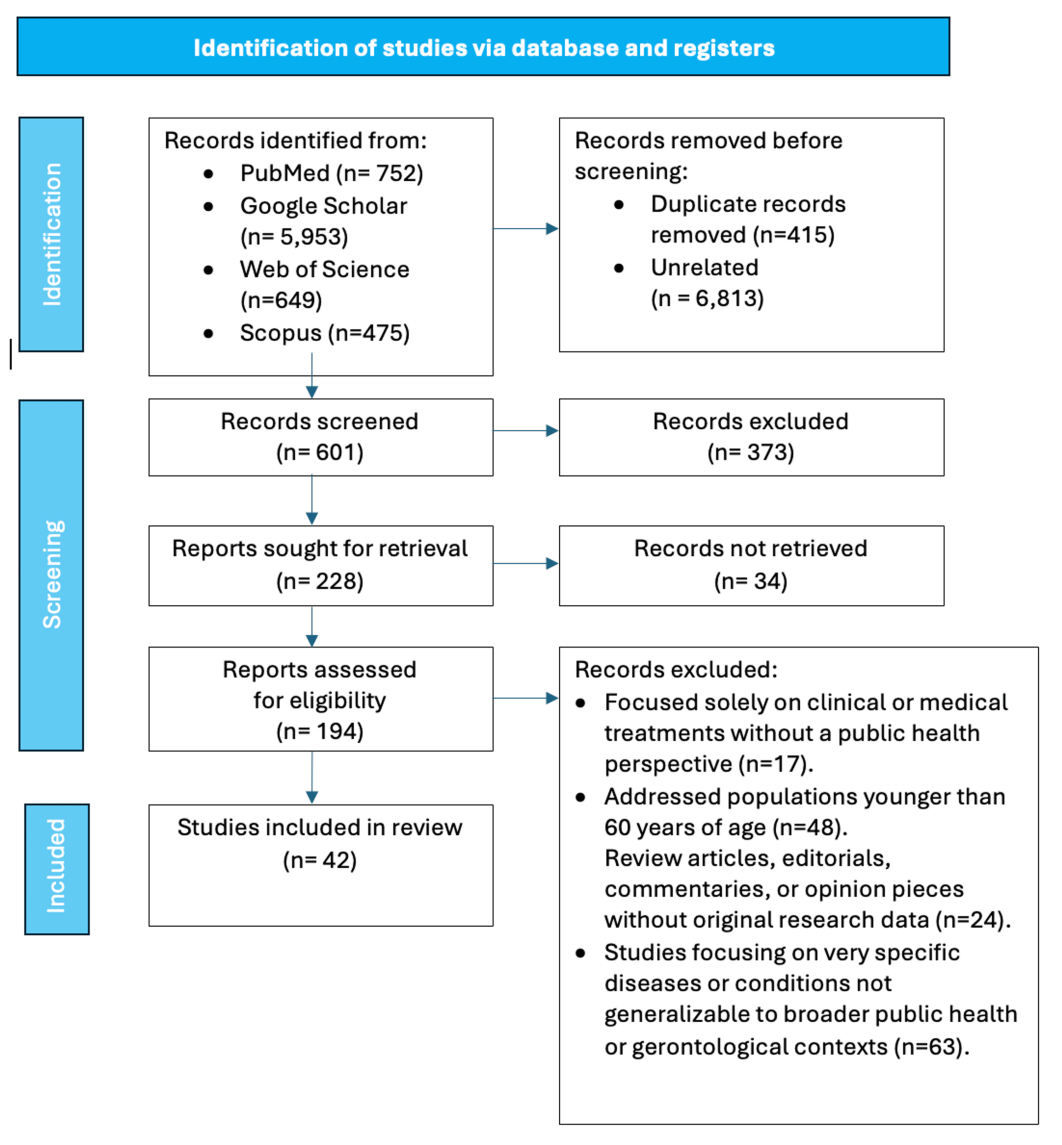
The process flow of the methodology of this scoping review. Image Credit: Nor Faiza Mohd. Tohit

Ethical approval was not required for this scoping review as it involved synthesizing already published data. However, ethical guidelines in conducting and reporting research were adhered to throughout the study. Figure 2 shows the process flow of this scoping review's methodology. By systematically mapping out the existing literature, this scoping review aims to provide a comprehensive overview of the integration of gerontology and public health, offering valuable insights for researchers, policymakers, and practitioners working to improve the health and well-being of older adults.

Review of literature

Themes and Trends in Current Perspectives on Gerontology in Public Health

The themes and trends in current perspectives on gerontology in public health reflect a comprehensive and inclusive approach to addressing the needs of the aging population. The main themes identified from the review are a) Aging demographics and public health implications and b) Attitudes and beliefs about aging and health. By focusing on the implications of aging demographics and shifting attitudes and beliefs about aging and health, public health strategies are evolving to promote healthy, active, and dignified aging for all individuals.

Aging demographics and public health implications:The aging demographics have undergone a significant transformation in the past decade, with substantial implications for public health. Globally, the aging population is increasing at an unprecedented rate. According to the WHO (2015), the number of individuals aged 65 and older is projected to double by 2050, reaching over 1.5 billion [1]. This demographic shift necessitates a re-evaluation of public health priorities and strategies to accommodate the diverse needs of older adults. One critical public health implication of aging demographics is the rising prevalence of chronic diseases and multi-morbidity among older adults. Chronic conditions such as heart disease, diabetes, and dementia are becoming increasingly common, leading to higher healthcare utilization and costs [3,9,21]. Public health initiatives gradually shift focus from disease management to integrated care models emphasizing prevention, early diagnosis, and coordinated care for chronic diseases [7,8,22]. This approach aims to enhance the quality of life for older adults and reduce the burden on healthcare systems. Another significant trend is the increasing awareness of the social determinants of health in the aging population. Income, education, housing, and social support are crucial in determining health outcomes among older adults [11,23,24]. Public health interventions are thus incorporating strategies to address these determinants, promoting health equity and inclusivity. For example, community-based programs focusing on improving social support and reducing isolation among older adults have shown promising results in enhancing mental and physical health outcomes [25,26]. Moreover, technological advancements are pivotal in shaping the public health landscape for older adults. Adopting digital health technologies, including telemedicine, wearable health monitoring devices, and electronic health records, is revolutionizing healthcare delivery to the aging population [18,19,27]. These technologies facilitate remote care, continuous health monitoring, and personalized interventions, making healthcare more accessible and efficient for older adults.

Attitudes and beliefs about aging and health:Attitudes and beliefs about aging significantly impact the implementation and effectiveness of public health policies and interventions. In recent years, there has been a noteworthy shift towards recognizing aging as a natural and positive part of life rather than solely focusing on its challenges and limitations. This paradigm shift is essential for promoting healthy aging and combating ageism, a pervasive issue in many societies. Ageism, defined as stereotypes, prejudice, and discrimination against individuals based on their age, has detrimental effects on the health and well-being of older adults [24,28]. Negative attitudes towards aging can lead to social isolation, reduced self-esteem, and poorer health outcomes [29]. Public health campaigns are increasingly addressing ageism by promoting positive images of aging and educating the public about the contributions and capabilities of older adults. Furthermore, there is a growing emphasis on the concept of "successful aging," which includes not only the absence of disease but also maintaining physical and cognitive function and engaging in meaningful social activity [30]. This holistic approach recognizes older adults' diverse experiences and needs and promotes strategies to enhance overall well-being throughout aging. Thus, Public health programs incorporate physical activity, cognitive training, and social engagement to support successful aging [31]. The belief in the importance of autonomy and independence among older adults has also gained prominence. Public health initiatives are increasingly designed to empower older adults to take control of their health and make informed decisions. This empowerment is facilitated through education, resource access, and supportive environments encouraging active and healthy lifestyles [32].

Public Health Interventions in Gerontology: A Decade of Progress

**Figure 3:**
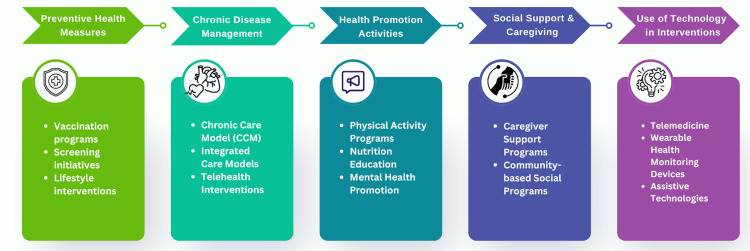
Public Health Interventions in Gerontology. Image Credit: Nor Faiza Mohd. Tohit.

Public health interventions for older adults have evolved significantly over the past decade, driven by advancements in preventive health measures, chronic disease management, health promotion activities, social support and caregiving programs, and the use of technology. As depicted in Figure 3, these interventions collectively aim to enhance the health and well-being of the aging population, addressing medical and social health determinants. Continued research and innovation in these areas are essential to meet the growing needs of older adults and ensure healthy aging for all.

Preventive health measures: Preventive health measures are cornerstone strategies in public health, particularly in the context of an aging population. Immunization, screening programs, and lifestyle interventions are critical to preventing diseases and promoting health among older adults [33-35]. Over the past decade, significant advancements have been made in these areas. Vaccination against influenza and pneumococcal infections continues to be a primary preventive measure. Studies have shown that vaccination significantly reduces morbidity and mortality among older adults [36]. Furthermore, increased efforts in promoting vaccinations through public health campaigns have led to higher immunization rates, thereby reducing the burden of preventable diseases. Screening programs for early detection of diseases such as cancer, diabetes, and cardiovascular conditions have also seen improvement [37,38]. Regular screenings facilitate early diagnosis and treatment, thereby improving health outcomes. For example, colonoscopy screenings have proven effective in detecting colorectal cancer at an early, treatable stage [39]. Lifestyle interventions, including diet and exercise programs, aim to prevent non-communicable diseases and promote overall health. The effectiveness of such interventions is well-documented. A systematic review by Marquez et al. (2020) highlighted that regular physical activity significantly reduces the risk of chronic diseases, improves physical functioning, and enhances quality of life in older adults [40].

Chronic disease management:Chronic disease management is a critical aspect of gerontological public health due to the high prevalence of chronic conditions among older adults. Effective management strategies involve comprehensive care models integrating medical, psychosocial, and behavioral interventions. One successful approach is the Chronic Care Model (CCM), which emphasizes the organization of healthcare services to improve the management of chronic diseases [41]. The CCM has been widely implemented and shown to enhance care coordination, patient self-management, and health outcomes in older adults with chronic conditions such as diabetes and heart disease. Integrated care models that combine healthcare delivery with social and community services are also gaining traction. These models provide holistic care that addresses both medical and social needs. For example, the Program of All-Inclusive Care for the Elderly (PACE) integrates medical care, social services, and long-term care for frail older adults (Boult et al., 2013) [42], significantly improving health outcomes and reducing hospitalizations [42,43]. Telehealth interventions have also become an essential part of chronic disease management. Telemonitoring programs allow healthcare providers to monitor patients' health status and provide timely interventions remotely. Studies have demonstrated that telehealth significantly improves clinical outcomes for chronic conditions such as hypertension and chronic obstructive pulmonary disease (COPD) [44,45].

Health promotion activities:Health promotion activities aim to empower older adults to take control of their health and adopt healthy behaviors. These diverse activities include initiatives focused on physical activity, nutrition, mental health, and fall prevention. Physical activity programs tailored for older adults have been shown to have numerous benefits, including improved cardiovascular health, improved cognitive function, increased muscle strength, and better balance, which reduces the risk of falls [14,15,46-48]. Community-based exercise programs such as Tai Chi classes and walking groups have effectively promoted physical activity among older adults. Nutrition education and counseling are also crucial components of health promotion. Programs encouraging healthy eating habits and improving nutritional knowledge can prevent malnutrition, obesity, and chronic conditions [15,49,50]. Mental health promotion is another critical area. Interventions that address depression, anxiety, and cognitive decline among older adults are essential for overall well-being. Cognitive training programs and social engagement activities have been found to maintain cognitive function and reduce the risk of dementia [51-53].

Social support and caregiving programs:Social support and caregiving programs play a vital role in the well-being of older adults, particularly those living with chronic conditions or disabilities. These programs aim to provide practical and emotional support to older adults and their caregivers. Caregiver support programs are designed to alleviate the burden on informal caregivers, often family members. Such programs offer education, respite care, and counseling to help caregivers manage their responsibilities and maintain their health. Evidence suggests that caregiver support programs improve the quality of life for caregivers and care recipients [54-56]. Community-based programs that foster social connections and reduce isolation among older adults are also essential. Social isolation has been linked to various adverse health outcomes, including increased mortality risk [9,57,58]. Programs that promote social interaction through group activities, volunteer opportunities, and community centers have been shown to improve mental and physical health outcomes [59,60].

Use of technology in interventions:Technological innovations have significantly transformed public health interventions for older adults. Technology offers tools to enhance healthcare delivery, monitor health conditions, and improve the quality of life for older adults. Telemedicine has gained widespread acceptance, particularly after the COVID-19 pandemic. Video consultations enable older adults to access healthcare services remotely, reducing the need for travel and minimizing exposure to infectious diseases. Telemedicine effectively manages chronic conditions, provides mental health services, and conducts routine health assessments [18,61-63]. Wearable health monitoring devices like fitness trackers and smartwatches allow continuous monitoring of vital signs and physical activity levels. These devices can detect early signs of health deterioration and prompt timely medical intervention. A study by Varnfield et al. (2014) demonstrated the efficacy of wearable devices in managing cardiovascular health and reducing hospital readmissions [64]. Assistive technologies, including smart home systems and mobility aids, promote independence and safety for older adults. Smart home systems equipped with sensors can monitor daily activities and alert caregivers or healthcare providers in case of emergencies, such as falls [13,64]. Mobility aids such as robotic exoskeletons enhance mobility and reduce the risk of injuries [27,65].

Identified Gaps in the Literature

Despite substantial advancements in the intersection of gerontology and public health, several critical gaps and challenges persist. Addressing these gaps is necessary for developing comprehensive strategies that effectively improve the health and well-being of older adults.

Limited inclusion of diverse populations:One significant gap in the existing literature is the underrepresentation of diverse populations. Many studies predominantly focus on Caucasian older adults, which limits the generalizability of findings to minority groups. Research on aging among racial and ethnic minorities, marginalized groups, and those from varied socioeconomic backgrounds is sparse. This lack of diversity in study populations overlooks the unique health challenges and cultural factors influencing these groups. Future research must prioritize inclusivity by encompassing a broader range of demographic groups to ensure that public health interventions are equitable and culturally competent [66].

Insufficient focus on mental health: While physical health interventions for older adults are well-researched, there is a notable deficiency in studies addressing mental health issues such as depression, anxiety, and cognitive decline. Mental health is integral to overall well-being, yet it remains inadequately explored in the context of public health for older adults. This gap underscores the need for comprehensive mental health interventions interwoven with physical health programs. Integrating mental health screenings and support services into routine geriatric care can help identify and address mental health issues early [67].

Economic barriers to accessing care: Economic barriers significantly challenge older adults' access to healthcare services. Limited research exists on the impact of financial constraints on the utilization of preventive health measures, chronic disease management programs, and health promotion activities. High out-of-pocket costs, lack of insurance coverage, and insufficient financial resources can deter older adults from seeking necessary care. Investigating the economic barriers and exploring policy solutions to reduce financial burdens is essential for creating accessible and affordable healthcare systems for aging populations [6,68,69].

Fragmented care systems: The fragmented nature of care systems often hampers the effective management of chronic diseases among older adults. Studies highlight the need for integrated care models coordinating services across various healthcare providers and settings. However, the practical implementation of such models is scant in the literature. Research should focus on developing and evaluating integrated care approaches that seamlessly connect primary care, specialty care, social services, and community resources. This integration can enhance continuity of care and improve health outcomes for older adults with complex health needs [70,71].

Lack of longitudinal data: Much of the existing research on public health interventions for older adults relies on cross-sectional data, which provides a snapshot of a specific point in time. This approach limits the understanding of the long-term effects and sustainability of interventions. Longitudinal studies that follow older adults over extended periods are crucial for assessing the enduring impact of preventive health measures, chronic disease management programs, and health promotion activities [72-74]. These studies can also shed light on changes in health status and healthcare needs as individuals age [75].

Underutilization of technology: Although there is growing interest in using technology to support health interventions for older adults, adopting and integrating such technologies remain limited. Research often focuses on the feasibility and acceptability of digital health tools without thoroughly evaluating their long-term effectiveness and scalability. There is a need for more rigorous studies that assess the impact of technologies like telehealth, health monitoring devices, and digital platforms on health outcomes, adherence to treatment, and quality of life among older adults. Additionally, addressing the digital divide by ensuring technology access and literacy is essential for maximizing the benefits of digital health interventions [76].

Policy and regulatory challenges: The literature reveals a gap in understanding the policy and regulatory frameworks that support or hinder public health interventions for older adults. Effective public health strategies require supportive policies that facilitate implementation and sustainability. Research should examine the impact of existing policies on the health of older adults and identify gaps that need to be addressed. Advocacy for policy changes, such as improved insurance coverage for preventive services and chronic disease management, is critical for enhancing the reach and effectiveness of public health interventions [77].

Environmental and community factors: Current research often overlooks the influence of environmental and community factors on the health and well-being of older adults. Safe housing, accessible transportation, and age-friendly community design substantially promote healthy aging [78]. Studies need to explore the impact of these environmental determinants and develop strategies to create supportive living environments for older adults. Community-based participatory research can also engage older adults in identifying and addressing local health challenges [79,80].

Interdisciplinary collaboration: Effective public health interventions for older adults require collaboration across various disciplines, including medicine, public health, social work, and allied health professions. However, the literature often lacks a focus on interdisciplinary approaches to geriatric care. Promoting multidisciplinary research and practice can facilitate more holistic and comprehensive care for older adults. Future studies should explore models of multidisciplinary collaboration and their impact on health outcomes and service delivery [81].

Cultural competence in intervention design: There is an evident gap in culturally competent public health interventions tailored to the diverse cultural backgrounds of older adults. Cultural competence involves understanding and respecting cultural differences and incorporating this knowledge into intervention design and delivery. Research should emphasize developing and evaluating culturally tailored interventions that address the unique needs and preferences of older adults from different cultural backgrounds. Training healthcare providers in cultural competence is also essential for delivering effective and respectful care [82,83].

The gerontology and public health literature gaps highlight the need for a more inclusive, comprehensive, and interdisciplinary approach to research and practice. Addressing these gaps will require concerted efforts to include diverse populations, integrate mental health with physical health, overcome economic and systemic barriers, utilize technology effectively, and develop supportive policies and environments. Future research should focus on long-term, culturally competent, and community-based interventions that promote healthy aging and improve the quality of life for all older adults.

Challenges in Implementing Public Health Interventions for Older Adults

Implementing public health interventions for older adults presents several challenges, including addressing complex health needs, overcoming resource constraints, adapting healthcare systems, leveraging technology, addressing social determinants of health, overcoming cultural barriers, and ensuring effective communication. By understanding and addressing these challenges, public health professionals can develop more effective and sustainable strategies to improve the health and well-being of older adults [43]. Table 2 summarizes the challenges in implementing public health interventions for older adults with the proposed solution [43].

**Table 2:** Summary of the challenges in implementing public health interventions for older adults with the proposed solution.

Areas of Concern	Description	Challenges	Solutions
1. Complex Health Needs and Multi-Morbidity	Older adults often suffer from multiple chronic conditions simultaneously (multi-morbidity), which complicates the implementation of public health interventions. Management of various conditions requires coordinated and comprehensive care approaches that address the diverse health needs of older adults.	Designing and delivering effective interventions across multiple chronic conditions while considering the interactions between various diseases and treatments.	Implement integrated care models that facilitate coordinated care across healthcare providers and settings. For example, the Program of All-Inclusive Care for the Elderly (PACE) offers a holistic approach to managing multi-morbidity in older adults (Boult et al., 2013).
2. Resource Constraints	Public health interventions for older adults often require significant resources, including funding, trained personnel, and infrastructure. Resource constraints can limit the reach and effectiveness of these interventions, particularly in low-resource settings.	Securing adequate funding and resources to implement and sustain comprehensive public health programs for older adults.	Leverage community resources and partnerships to augment available resources. Community health workers and volunteer programs can play a critical role in extending the reach of public health interventions.
3. Healthcare System Limitations	Existing healthcare systems are often not fully equipped to meet the needs of an aging population. Many healthcare systems are designed to address acute care needs rather than chronic and long-term care, which are more pertinent to older adults.	Adapting healthcare systems to provide continuous, long-term care that addresses older adults' chronic conditions and functional limitations.	Reform healthcare policies to prioritize geriatric care and allocate appropriate resources for chronic disease management and preventive care. Implement care models that integrate medical care with social services to address the comprehensive needs of older adults.
4. Technological Barriers	While technology can significantly enhance public health interventions, older adults often face barriers to technology adoption due to lack of familiarity, cognitive decline, and physical limitations.	Ensuring that older adults can use and benefit from technological interventions to improve their health and well-being.	Provide training and support to older adults to familiarize them with new technologies. Design user-friendly technologies that accommodate the physical and cognitive capabilities of older adults. Engage older adults in designing and testing these technologies to ensure their needs and preferences are met.
5. Social Determinants of Health	Social determinants such as socioeconomic status, education, housing, and social support significantly influence the health outcomes of older adults. Addressing these determinants is essential for the success of public health interventions.	Implementing interventions that effectively address the broad range of social determinants affecting the health of older adults.	Develop multifaceted interventions that address both health and social needs. Programs that include housing support, income assistance, and opportunities for social engagement can improve the overall well-being of older adults.
6. Cultural and Attitudinal Barriers	Societal attitudes towards aging and cultural beliefs can influence the acceptance and success of public health interventions. Ageism and negative stereotypes about aging can hinder the implementation of effective programs.	Overcoming negative attitudes and misconceptions about aging can discourage older adults from participating in public health programs.	Promote positive attitudes towards aging through public health campaigns and education. Engage older adults in planning and developing interventions to ensure they are culturally sensitive and acceptable.
7. Communication and Outreach Limitations	Effective communication is critical for the success of public health interventions. However, reaching older adults, especially those isolated or with sensory impairments, can be challenging.	Ensuring that health information reaches all older adults, including those who are socially isolated or have communication impairments.	To disseminate health information, utilize multiple communication channels, including print, digital, and in-person outreach. Develop materials that are accessible and easy to understand, and provide assistive devices as needed.

Implications for Public Health Policy and Practice

The demographic shift towards an aging population has profound implications for public health policy and practice. As the proportion of older adults increases, public health systems must adapt to meet their unique needs.

Aging population and health system capacity: The increasing number of older adults necessitates re-evaluating health system capacity and resource allocation. Policies must focus on expanding geriatric care services, including training healthcare professionals, increasing the number of geriatric specialists, and integrating geriatric principles into primary healthcare [1,4,84]. Additionally, policies that support the creation and expansion of age-friendly health infrastructures, such as easily accessible healthcare facilities equipped to address the health needs of older adults [85], are needed.

Chronic disease management: Effective chronic disease management is critical as the prevalence of chronic conditions such as diabetes, cardiovascular diseases, and arthritis is higher among older adults. Policies should promote integrated care models that provide coordinated and continuous care, leveraging medical and social services. This includes fostering collaborations between various sectors, such as healthcare providers, social services, community organizations, and family caregivers [86-88].

Preventive health measures: Preventive health measures, including vaccinations, screenings, and lifestyle interventions, must be prioritized in public health policy. Policies should ensure the availability and accessibility of preventive services to all older adults, particularly those from marginalized communities. Public health campaigns to increase awareness and uptake of preventive services can play a vital role in improving health outcomes and reducing healthcare costs associated with chronic diseases [89-91].

Social determinants of health: Addressing the social determinants of health, such as income, education, housing, and social support, is crucial for promoting health equity among older adults. Policies should aim to reduce health disparities by ensuring that older adults can access the resources they need to lead healthy and fulfilling lives. This includes affordable housing, financial security, access to nutritious food, and opportunities for social engagement [92-94].

Technological integration:Supportive policies must facilitate the integration of technology into healthcare services. This includes investing in telehealth infrastructure, ensuring digital literacy among older adults, and addressing the digital divide to ensure equitable access to technological innovations. Policies should also support developing and implementing assistive technologies that enhance older adults' quality of life and independence [95].

Recommendations for Public Health Practitioners

**Figure 4:**
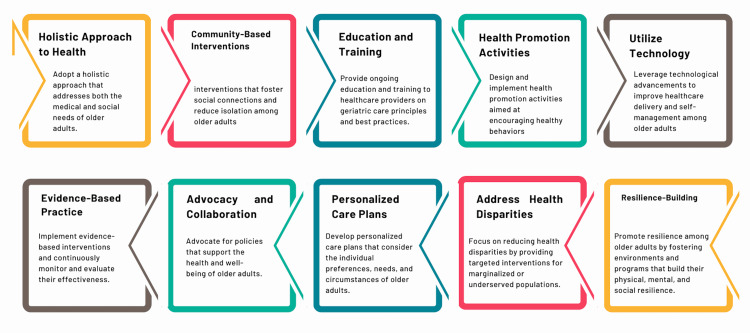
Recommendations for public health practitioners. Image Credit: Nor Faiza Mohd. Tohit

Public health practitioners play a critical role in translating policy into practice and implementing interventions that improve the health and well-being of older adults. The following recommendations in Figure 4 can guide these efforts. By adopting these recommendations, public health practitioners can effectively implement interventions that address the diverse needs of older adults. Such efforts ensure that aging populations can lead healthy, active, and fulfilling lives, ultimately contributing to society's well-being.

Strengths and Limitations of the Scoping Review

**Figure 5:**
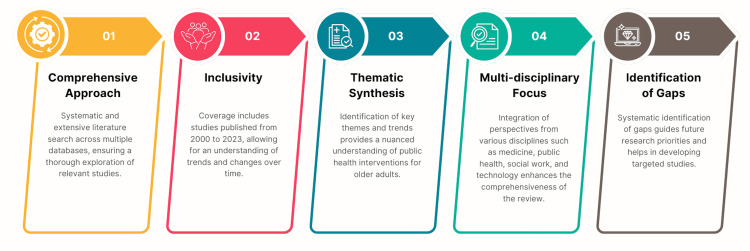
Strength of the Scoping Review. Image Credit: Nor Faiza Mohd. Tohit

**Figure 6:**
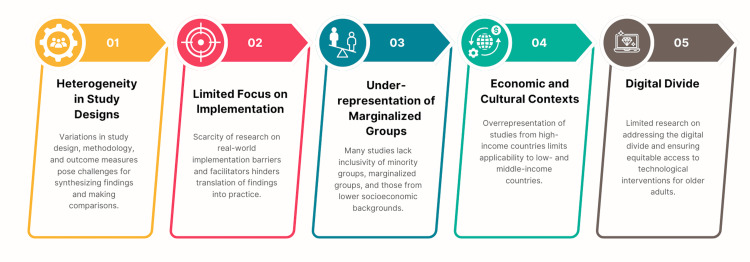
Limitations of the scoping review. Image Credit: Nor Faiza Mohd. Tohit

**Figure 7:**
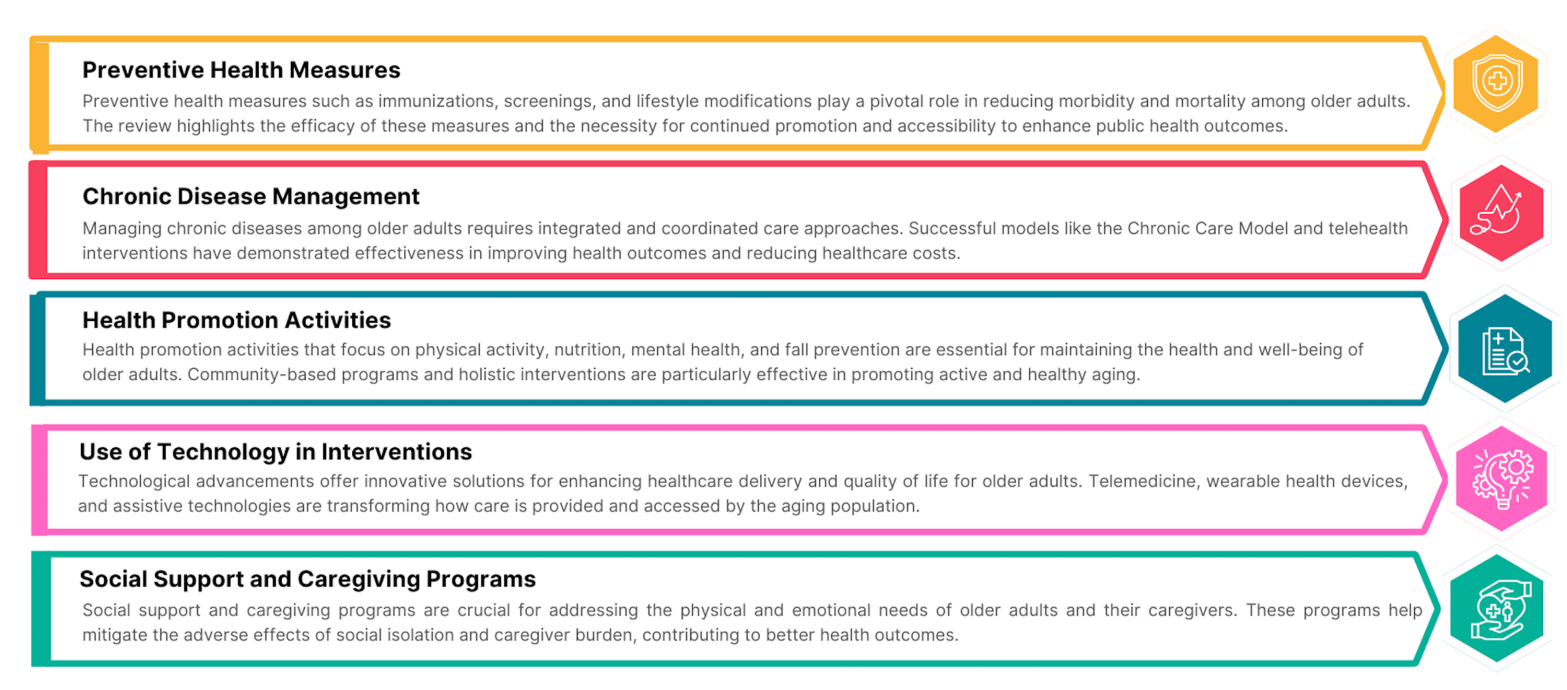
The summary of this scoping review is illustrated in this figure.

A thorough evaluation of this scoping review's strengths (Figure 5) and limitations (Figure 6) provides valuable insights into its overall robustness and areas needing improvement. On one hand, the review's systematic and comprehensive approach ensures an extensive exploration of the literature, covering a broad timeframe and incorporating diverse disciplinary perspectives. This inclusivity allows for a nuanced understanding of public health interventions for older adults. However, the review also faces methodological challenges, such as heterogeneity in study designs, underrepresentation of marginalized groups, and limited focus on real-world implementation. These limitations highlight the need for ongoing efforts to address gaps in the literature and enhance the effectiveness and generalizability of future research in gerontological public health. The summary of this scoping review is illustrated in Figure 7.

Areas for Further Research

The scoping review highlights significant gaps and challenges in gerontology and public health, suggesting several critical areas for further research. Advancing research in these identified areas (Table 3) can significantly enhance our understanding and effectiveness of public health interventions for older adults. Researchers and practitioners can develop more targeted, inclusive, and sustainable public health strategies by addressing diversity, mental health, economic barriers, integrated care, technology, cultural competence, social determinants, interdisciplinary collaboration, and implementation science [76,96-99]. Ultimately, these efforts will improve the health and well-being of the aging population, ensuring that older adults can lead healthy, active, and fulfilling lives.

**Table 3:** Areas for further research.

Area	Focus	Rationale
1. Diversity and Inclusivity in Research	Investigating the health needs and outcomes of diverse populations, including racial and ethnic minorities, marginalized older adults, and individuals from varying socioeconomic backgrounds.	Many existing studies predominantly focus on Caucasian older adults, limiting the generalizability of findings to other demographic groups. Future research can identify unique health challenges and solutions pertinent to diverse populations by including a broader spectrum of participants.
2. Mental Health Interventions	Developing and evaluating mental health interventions tailored for older adults, addressing conditions such as depression, anxiety, and cognitive decline.	While physical health is often the focus of public health interventions, mental health is equally crucial for the overall well-being of older adults. Integrating mental health care into public health programs can improve holistic health outcomes.
3. Longitudinal Studies	Conducting longitudinal research to assess the long-term effects and sustainability of public health interventions for older adults.	Cross-sectional studies provide only a snapshot of health outcomes at a specific time. Longitudinal studies can offer insights into the enduring impact of interventions, changes in health status, and the evolving needs of older adults.
4. Economic Barriers and Policy Solutions	Investigating the economic barriers that older adults face in accessing healthcare services and exploring policy solutions to mitigate these challenges.	Financial constraints can prevent older adults from utilizing necessary healthcare services. Understanding these barriers and developing supportive policies can improve access to care and health outcomes.
5. Integrated Care Models	Developing and evaluating integrated care models that coordinate services across healthcare providers, social services, and community resources.	Fragmented care systems often hinder effective chronic disease management and preventive care for older adults. Integrated care models can enhance care continuity and address medical and social needs.
6. Technological Innovations	Assessing the long-term effectiveness and scalability of technological innovations in public health interventions for older adults.	While technology has great potential to enhance healthcare delivery, its long-term impact and feasibility must be thoroughly evaluated. Ensuring that older adults can effectively use and benefit from these technologies is crucial.
7. Cultural Competence in Intervention Design	Developing and evaluating culturally competent public health interventions tailored to the diverse backgrounds of older adults.	Cultural competence involves understanding and respecting cultural differences and incorporating this knowledge into intervention design. Tailored interventions can improve acceptance and effectiveness.
8. Social Determinants of Health	Exploring the impact of social determinants such as housing, education, income, and social support on the health of older adults and developing interventions that address these factors.	Social determinants play a crucial role in health outcomes. Interventions that address these broader factors can promote health equity and improve well-being.
9. Interdisciplinary Collaboration	Promoting interdisciplinary research and practice that involves collaboration across various fields such as medicine, public health, social work, and technology.	Effective public health interventions for older adults require a multidisciplinary approach. Collaborating across disciplines can lead to more comprehensive and holistic care strategies.
10. Implementation Science	Studying the real-world implementation of public health interventions, identifying barriers and facilitators to implementation, and developing strategies to enhance scalability and sustainability.	Moving from theory to practice involves numerous challenges. Understanding these challenges through implementation science can improve the uptake and effectiveness of interventions.

## Conclusions

In conclusion, while significant strides have been made in gerontological public health, ongoing research, policy reform, and practice improvements are necessary to meet the evolving needs of the aging population. By addressing the identified gaps and challenges and implementing evidence-based, inclusive, and sustainable interventions, we can improve the health and well-being of older adults globally. This comprehensive approach will ensure that older adults receive the care and support they need to lead healthy, active, and dignified lives.
